# Efficacy, safety and tolerability of GSK2190915, a 5-lipoxygenase activating protein inhibitor, in adults and adolescents with persistent asthma: a randomised dose-ranging study

**DOI:** 10.1186/1465-9921-14-54

**Published:** 2013-05-17

**Authors:** Richard MA Follows, Neil G Snowise, Shu-Yen Ho, Claire L Ambery, Kevin Smart, Barbara A McQuade

**Affiliations:** 1 GlaxoSmithKline, Stockley Park West, Uxbridge, Middlesex UB11 1BT, UK; 2 GlaxoSmithKline, 5 Moore Drive, Research Triangle Park, NC 2770, USA; 3 GlaxoSmithKline, Gunnels Wood Road, Stevenage Herts, SG1 2NY, UK; 4 Current address: Vectura Group plc, One Prospect West, Chippenham, Wiltshire SN14 6FH, UK; 5 Current address: Roche Products Limited, 6 Falcon Way, Shire Park, Welwyn Garden City AL7 1TW, UK

**Keywords:** GSK2190915, FLAP, Asthma, Efficacy, Safety, Tolerability, Dose-ranging

## Abstract

**Background:**

GSK2190915 is a high affinity 5-lipoxygenase-activating protein inhibitor being developed for the treatment of asthma. The objective of this study was to evaluate GSK2190915 efficacy, dose–response and safety in subjects with persistent asthma treated with short-acting beta2-agonists (SABAs) only.

**Methods:**

Eight-week multicentre, randomised, double-blind, double-dummy, stratified (by age and smoking status), parallel-group, placebo-controlled study in subjects aged ≥12 years with a forced expiratory volume in 1 second (FEV_1_) of 50–85% predicted. Subjects (n = 700) were randomised to receive once-daily (QD) oral GSK2190915 (10–300 mg), twice-daily inhaled fluticasone propionate 100 μg, oral montelukast 10 mg QD or placebo. The primary endpoint was mean change from baseline (randomisation) in trough (morning pre-dose and pre-rescue bronchodilator) FEV_1_ at the end of the 8-week treatment period. Secondary endpoints included morning and evening peak expiratory flow, symptom-free days and nights, rescue-free days and nights, day and night-time symptom scores, day and night-time rescue medication use, withdrawals due to lack of efficacy, Asthma Control Questionnaire and Asthma Quality of Life Questionnaire scores.

**Results:**

For the primary endpoint, there was no statistically significant difference between any dose of GSK2190915 QD and placebo. However, repeated measures sensitivity analysis demonstrated nominal statistical significance for GSK2190915 30 mg QD compared with placebo (mean difference: 0.115 L [95% confidence interval: 0.00, 0.23], p = 0.044); no nominally statistically significant differences were observed with any of the other doses. For the secondary endpoints, decreases were observed in day-time symptom scores and day-time SABA use for GSK2190915 30 mg QD versus placebo (p ≤ 0.05). No dose–response relationship was observed for the primary and secondary endpoints across the GSK2190915 dose range studied; the 10 mg dose appeared to be sub-optimal. GSK2190915 was associated with a dose-dependent reduction in urinary leukotriene E_4_. The profile and incidence of adverse events were similar between treatment groups.

**Conclusion:**

Efficacy was demonstrated for GSK2190915 30 mg compared with placebo in day-time symptom scores and day-time SABA use. No additional improvement on efficacy endpoints was gained by administration of GSK2190915 doses greater than 30 mg. GSK2190915 was well-tolerated. These results may support further studies with GSK2190915 30 mg.

**Trial registration:**

Clinicaltrials.gov: NCT01147744.

## Background

Leukotrienes (LTs) are produced by mast cells, eosinophils, macrophages and neutrophils in response to allergic or inflammatory stimuli [1-3]. They are products of the 5-lipoxygenase (5-LO) pathway of arachidonic acid metabolism and their synthesis is initiated by 5-LO in concert with 5-lipoxygenase-activating protein (FLAP). This part of the pathway divides into two branches: one leading to production of LTB_4_ and the other to the production of the cysteinyl LTs (cysLTs) LTC_4_, LTD_4_ and LTE_4_, so-called because they contain the amino acid cysteine conjugated to their lipid backbone. The central role of LTs in asthma pathogenesis is well-established as they function as potent mediators of inflammation and airway constriction [4-7]. Concentrations of the products of the 5-LO pathway are increased in patients with asthma of all severities despite treatment with corticosteroids [8-10], the cornerstone anti-inflammatory therapy in asthma.

Leukotriene B_4_ is a potent chemoattractant of neutrophils [11]. Irrespective of therapy with corticosteroids, LTB_4_ concentrations were increased in the bronchoalveolar fluid, sputum and tissue of patients with severe asthma compared with non-asthmatic control subjects and patients with mild-moderate asthma [10,12]. Neutrophil numbers have been shown to increase in patients with severe asthma compared with milder asthma phenotypes, and neutrophilic inflammation is resistant to the effects of corticosteroids [9,12,13]. The cysLTs are potent constrictors of airway smooth muscle, they increase vascular permeability and serve as chemoattractants for eosinophils [4-6]. Cysteinyl LTs are directly involved in bronchoconstriction, airway oedema and mucus secretion, characteristic of the asthmatic phenotype [14]. Inhibition of cysLTs by the administration of the cysLT receptor antagonist montelukast elicits bronchodilatory effects and significantly reduces eosinophil numbers in the peripheral blood and induced sputum of patients with asthma [15]. In addition, administration of the 5-LO inhibitor zileuton to patients with severe asthma led to improvements in lung function [16]. These observations indicate that inhibition of the production of LTs via the two branches of the 5-LO pathway could confer beneficial effects on patients with persistent asthma.

GSK2190915 is a high affinity FLAP inhibitor that attenuates the production of LTs through inhibition of the first step of the 5-LO pathway. *In vitro* and *in vivo* studies demonstrated that GSK2190915 reproducibly inhibited the production of both LTB_4_ and cysLTs [17]. In healthy subjects, GSK2190915 was well-tolerated with a systemic exposure that increased in a dose-related manner [18]. GSK2190915 also demonstrated dose-dependent inhibition of blood LTB_4_ production and of urinary excretion of the cysLT, LTE_4_[18]. The primary objective of the current study was to evaluate the efficacy, dose–response, safety and tolerability of GSK2190915 administered once-daily (QD) over an 8-week period in adolescents and adults with persistent uncontrolled asthma receiving a short-acting beta2-agonist (SABA). The secondary objective of this study was to explore the efficacy of GSK2190915 against established asthma treatments, namely montelukast and the inhaled corticosteroid (ICS) fluticasone propionate (FP).

## Methods

### Subjects

Subjects aged 12 years or older were eligible for enrolment if they had a diagnosis of asthma (as defined by the National Institutes of Health [19]) with a best (the highest of three technically acceptable measurements) pre-bronchodilator forced expiratory volume in 1 second (FEV_1_) of 50–85% of the predicted normal value, and reversibility of at least 12% and 200 mL within 30 minutes after inhaled salbutamol/albuterol. The original protocol also allowed males to be recruited to the study. However, the protocol was amended to include only females after findings of testicular toxicity in rats at high exposures of GSK2190915 following 6-month dosing were reported during the conduct of the study. Former and current smokers, with a smoking history of ≤10 pack years, were required to demonstrate a post-salbutamol/albuterol FEV_1_/forced vital capacity ratio of >0.70 to exclude subjects with fixed airways. Eligible subjects were required to have been taking a SABA for at least 3 months before screening. They were also required to be able to replace their SABA with salbutamol/albuterol to be used as rescue medication during the run-in and treatment periods. Other permitted medications included stable-dose immunotherapy, intranasal corticosteroids and short and long-acting antihistamines.

Exclusion criteria at screening included a history of life-threatening asthma (defined as an asthmatic episode that had required intubation and/or was associated with hypercapnoea, respiratory arrest or hypoxic seizures within the last 5 years), an asthma exacerbation requiring oral corticosteroids in the 3 months prior to screening or hospitalisation for asthma in the previous 6 months, an unresolved infection in the past 4 weeks leading to a change in asthma management or that affected the subject’s asthma status or ability to participate in the study, use of ICSs in the past 6 weeks or of systemic, oral or depot corticosteroids in the past 12 weeks. Non-smoking subjects were not permitted to have used tobacco products within 6 months of screening. Subjects were also excluded if they had received statins or other organic anion transport protein 1B1 substrates within 4 weeks of screening or had any adverse reaction including immediate or delayed hypersensitivity to any beta2-agonist, sympathomimetic drug, intranasal, inhaled or systemic corticosteroid.

### Study design

This was a Phase IIb, multicentre, randomised, double-blind, double-dummy, parallel-group, placebo-controlled study that was conducted from January 2010 to October 2011 at 89 sites in six countries (GlaxoSmithKline protocol: LPA112186; Clinicaltrials.gov registration number: NCT01147744). All subjects provided signed informed consent prior to screening. Local Ethics Review Committees provided approval for the study, which was conducted in accordance with Good Clinical Practice, applicable country-specific requirements and the guiding principles of the Declaration of Helsinki (2008).

Eligible subjects completed a 2-week pre-treatment run-in period during which they were required to attend the clinical unit for assessment of their adherence with an electronic daily diary (eDiary, AM3 device by ERT, Bavaria, Germany). At the end of the study run-in period, subjects were eligible to enter the treatment period if they had a morning pre-dose FEV_1_ between 50% and 85% of their predicted normal and had either any combination of the daily asthma symptom scores (day-time plus night-time) of ≥1, or used salbutamol/albuterol on at least 4 days of the last 7 consecutive days of the run-in period.

Subjects were randomised to receive GSK2190915 tablets (10 mg, 30 mg, 100 mg, 300 mg) QD (morning), FP 100 μg twice-daily (BID; morning and evening) via a Diskus™/Accuhaler™ (GlaxoSmithKline, Ware, UK) and montelukast 10 mg capsules QD (evening). A double dummy design was used to ensure blinding to treatment (see Additional file 1). Subjects received treatment on an out-patient basis for 8 weeks. Subjects aged 12–14 years were not randomised to the montelukast arm as the 10 mg dose is not licensed for this age group. Randomisation was stratified according to subject age and smoking status. Follow-up took place 1 week after completion of the treatment period. Following their withdrawal, all male subjects were offered a clinical assessment.

Subjects were withdrawn from study medication due to ‘lack of efficacy’ if they changed their asthma medication, had an asthma exacerbation (defined as worsening asthma requiring any treatment other than rescue salbutamol/albuterol) or had signs of asthma instability (defined as a fall in clinic FEV_1_ to <80% of value at randomisation, peak expiratory flow [PEF] < 80% of the mean run-in value on more than 3 days between consecutive visits or use of salbutamol/albuterol ≥12 inhalations/day on more than 2 days between consecutive visits).

Adherence to treatment was assessed from Week 4 until Week 8 (or early withdrawal) by counting returned tablets, capsules and reviewing the dosing counter on the Diskus/Accuhaler. Allocation to treatment group was determined according to a computer generated schedule; numbered containers were used to implement allocation. Neither the subject nor the investigator knew which study medication the subject was receiving.

### Efficacy assessments

Assessments of efficacy were based on measures of spirometry, including trough FEV_1_ (pre-bronchodilator and pre-dose); parameters recorded in the daily diary including symptoms, use of rescue medication and daily PEF; questionnaires including Asthma Control Questionnaire (ACQ)-6 and the Asthma Quality of Life Questionnaire (AQLQ) (+12).

The primary endpoint of the study was mean change from baseline (randomisation) in trough (morning pre-dose and pre-rescue bronchodilator) FEV_1_ at the end of the 8-week treatment period. Changes in FEV_1_ at weeks 1, 2, 4 and 6 were also assessed. Efficacy measures for secondary endpoints recorded by the patients using the daily diary were: mean change from baseline in daily trough (pre-dose and pre-rescue bronchodilator) morning and evening PEF averaged over the 8-week treatment period; mean change from baseline in the percentage of symptom-free days and nights and rescue-free days and nights; mean change from baseline in day and night-time symptom scores; mean change from baseline in day and night-time rescue salbutamol/albuterol use; withdrawals due to lack of efficacy and mean change from baseline in ACQ and AQLQ scores. In addition, other endpoints were assessed: symptom-free 24-hour periods, rescue-free 24-hour periods, 24-hour period symptom scores and 24-hour SABA use.

All post-randomisation FEV_1_ measurements were taken within 1 hour of the time FEV_1_ was measured at randomisation. Before attending the clinical unit for lung function assessments, subjects had to withhold their rescue medication for at least 6 hours. At weeks 1, 2, 4, 6 and 8, FEV_1_ was measured approximately 24 hours post-morning dose and approximately 12 hours post-evening dose.

### Pharmacokinetic/pharmacodynamic assessments

Blood samples for pharmacokinetic analysis were collected at pre-dose on weeks 0, 2 and 4. Post-dose samples were collected on weeks 1 and 8 within the following time windows: first sample: 0.5–2 hours; second sample: 2–5 hours. Plasma samples from subjects who received only GSK2190915 were analysed using a validated analytical method based on protein precipitation followed by high performance liquid chromatography with tandem mass spectrometry analysis. The lower limit of quantification for GSK2190915 was 5 ng/mL and the higher limit of quantification was 5000 ng/mL.

Three spot urine samples were collected at Week 0 and pre-dose on weeks 1 and 8 to determine mean change from baseline in urinary LTE_4_. Urine samples were analysed for LTE_4_ using a validated analytical method based on solid-phase extraction followed by high performance liquid chromatography with tandem mass spectrometric detection. The lower limit of quantification for LTE_4_ was 5 pg/mL and the higher limit of quantification was 1000 pg/mL.

### Safety and tolerability measurements

Safety and tolerability were assessed by monitoring adverse events during the treatment period until follow-up, and serious adverse events from screening to follow-up. Adverse events were coded using the Medical Dictionary for Regulatory Activities (MedDRA). Asthma exacerbations were recorded. Assessments of liver function, standard laboratory parameters, vital signs (pulse rate and systolic and diastolic blood pressure) and 12-lead electrocardiograms (ECGs) were also performed.

### Statistical analysis

The primary hypothesis of the study was that FEV_1_ in the GSK2190915 groups would demonstrate a significant increase at the end of the 8-week treatment period compared with that in the placebo group. Assuming a between-subject common standard deviation in FEV_1_ of 415 mL, it was estimated that it would be necessary to recruit 630 subjects (90 per group) to provide 89% power to detect a difference (two-sided 5% level) of 200 mL in pairwise comparisons between any GSK2190915 dose group and placebo.

A sequential testing procedure was followed to account for multiplicity from multiple pairwise comparisons: statistical comparison of the highest GSK2190915 dose with placebo was performed first and subsequent comparisons at lower doses continued in a sequential manner. A 5% level of statistical significance was claimed only if the preceding comparison was significant at the 5% level. Any other test results were not adjusted for multiplicity and any p-value ≤0.05 from those testing results was interpreted as nominally significant. Primary efficacy analysis was performed using analysis of covariance (ANCOVA) with a last observation carried forward approach used to impute missing data; repeat measures model was also performed for sensitivity analysis.

Secondary efficacy analysis was performed using ANCOVA, except for the comparison between each treatment group and placebo for the number of withdrawals due to lack of efficacy in which a Fisher’s Exact test was used. No multiplicity adjustments were made on the secondary efficacy endpoints. Any p value ≤0.05 was identified as nominally significant. The comparisons between active treatments were only for exploratory purposes. Population pharmacokinetic analysis of sparse GSK2190915 plasma concentration-time data was performed using non-linear mixed effects modelling as was the population pharmacokinetic-pharmacodynamic analysis to assess the plasma GSK2190915 concentration versus urinary LTE_4_ relationship. Non-quantifiable pharmacokinetic and pharmacodynamic data were treated as missing for the population analysis. No formal statistical analysis was performed on safety data. For all endpoints, data are reported for subjects who received at least one dose of study medication.

## Results

### Subjects

**Figure 1:**
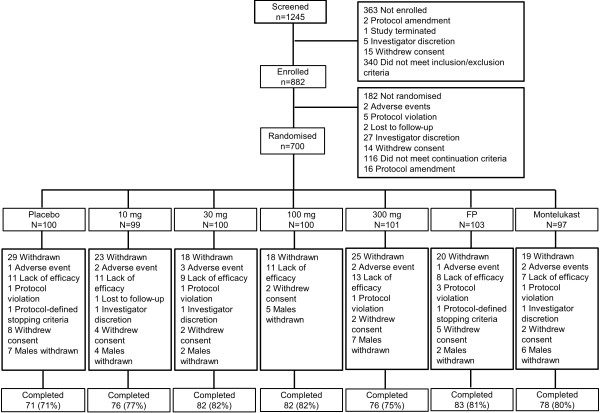
**CONSORT diagram.** Subject flow through study. FP = fluticasone propionate.

Of 1245 subjects screened, 700 were randomised and 548 completed the study (Figure 1). One hundred and thirteen (16%) subjects, including 56 males, were randomised under the original protocol. However, after the protocol was amended to withdraw males and include females only, an additional 587 (84%) female subjects were subsequently recruited. One hundred and fifty-two (22%) subjects withdrew from the study and the most frequent reasons for withdrawal were: lack of efficacy (70 subjects), decision to withdraw all male subjects (33 subjects) and withdrawal of consent (25 subjects). The numbers of subjects in each group withdrawing from the study were similar. Demographic and baseline subject characteristics were well-matched across treatment groups (Table 1).

**Table 1:** Demographic and baseline subject characteristics

	**Placebo**	**GSK2190915 10 mg QD**	**GSK2190915 30 mg QD**	**GSK2190915 100 mg QD**	**GSK2190915 300 mg QD**	**FP 100 μg BID**	**Montelukast 10 mg QD**
	**N = 100**	**N = 99**	**N = 100**	**N = 100**	**N = 101**	**N = 103**	**N = 97**
	**n (%)**	**n (%)**	**n (%)**	**n (%)**	**n (%)**	**n (%)**	**n (%)**
Age in years; Mean [range]	42.3 [12–71]	40.0 [12–75]	43.1 [12–73]	42.2 [12–69]	42.2 [12–69]	41.5 [12–74]	44.3 [15–78]
BMI in kg/m^2^; Mean [range]	27.5 [16–47]	27.5 [17–49]	26.8 [16–43]	27.8 [16–52]	26.9 [19–63]	27.0 [15–55]	27.1 [18–54]
Sex; n (%)							
Female	88 (88)	91 (92)	94 (94)	92 (92)	93 (92)	97 (94)	89 (92)
Male	12 (12)	8 (8)	6 (6)	8 (8)	8 (8)	6 (6)	8 (8)
Race; n (%)							
White	82 (82)	74 (75)	78 (78)	78 (78)	78 (77)	83 (81)	76 (78)
Black	6 (6)	12 (12)	10 (10)	9 (9)	9 (9)	7 (7)	9 (9)
Other	12 (12)	13 (13)	12 (12)	13 (13)	14 (14)	13 (13)	12 (12)
Smoking-history; n (%)							
Never smoked	78 (78)	85 (86)	83 (83)	77 (77)	86 (85)	86 (83)	78 (80)
Current smoker	12 (12)	9 (9)	10 (10)	11 (11)	10 (10)	13 (13)	13 (13)
Former smoker	10 (10)	5 (5)	7 (7)	12 (12)	5 (5)	4 (4)	6 (6)
FEV_1_ in L; Mean [range]	2.0 [1.2–3.7]	2.0 [1.0–3.3]	1.9 [1.1–2.8]	2.0 [1.0–3.5]	2.0 [1.1–3.4]	2.0 [1.0–4.0]	2.0 [0.9–3.5]
% predicted; Mean [range]	68.7 [51–87]	67.7 [50–96]	66.1 [50–85]	67.8 [51–85]	66.8 [51–85]	66.6 [37–85]	69.2 [50–85]
Atopy, n%	22 (22)	30 (30)	31 (31)	31 (31)	35 (35)	35 (34)	34 (35)

*QD* Once-daily, *FP* Fluticasone propionate, *BID* Twice-daily, *BMI* Body mass index, *FEV*_*1*_ Forced expiratory volume in 1 second.

### Efficacy

**Figure 2:**
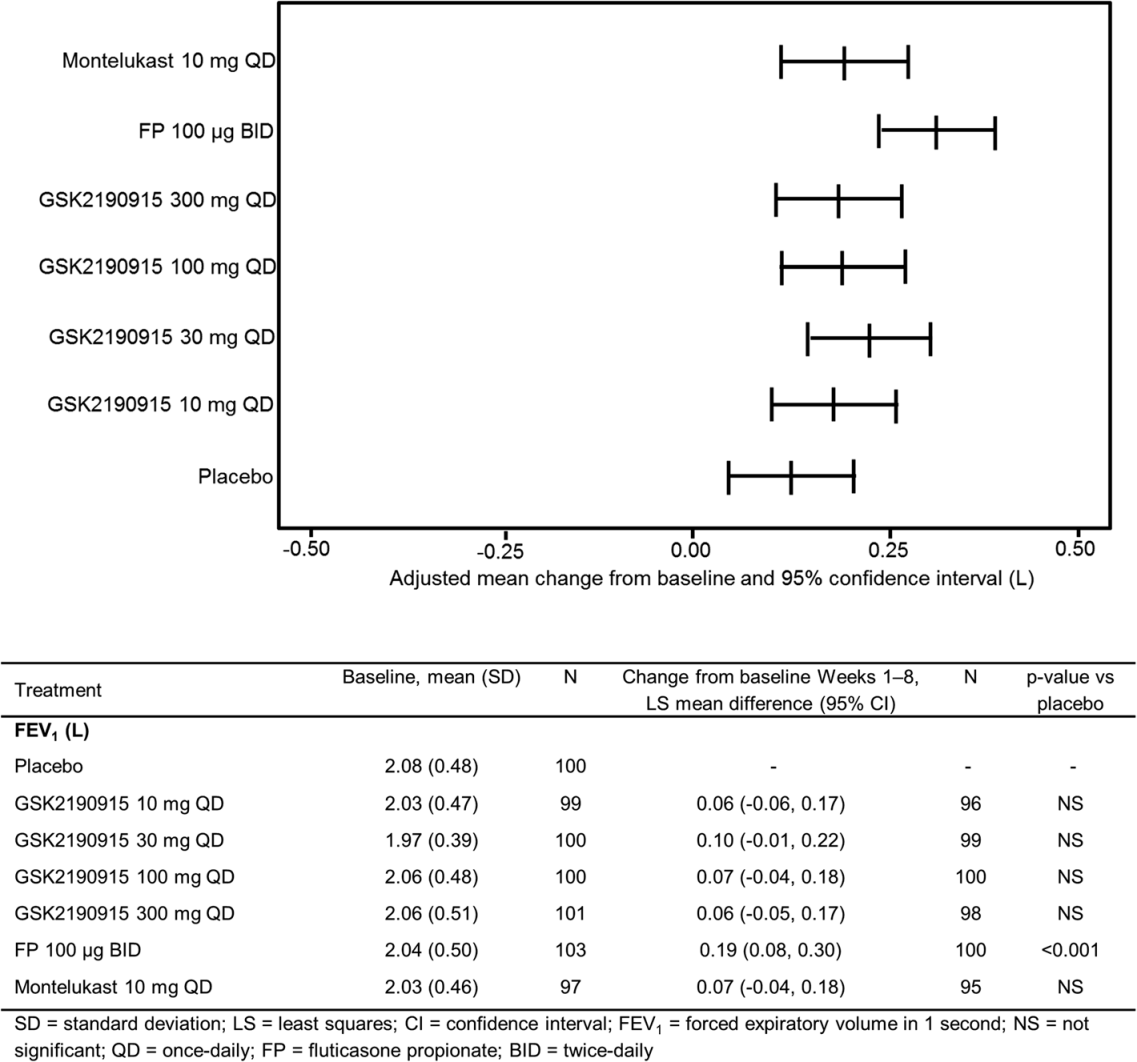
**Primary efficacy endpoint.** Plot of adjusted mean change from baseline in trough FEV_1_ at Week 8 and summary of statistical analysis.

**Figure 3:**
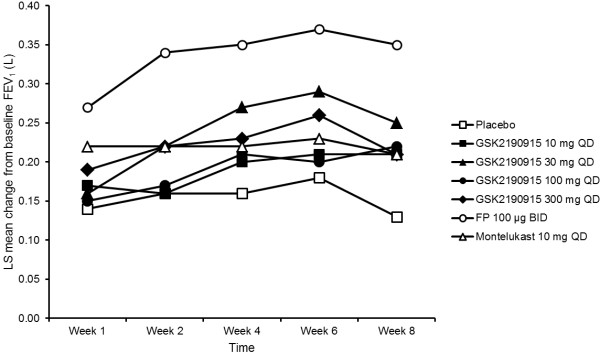
**Sensitivity analysis of primary efficacy endpoint.** Repeated measures analysis of change from baseline in trough FEV_1_ (L). LS = least squares.

At the end of the 8-week treatment period, there was no statistically significant difference between any dose of GSK2190915 QD and placebo for mean change from baseline in trough (morning pre-dose and pre-rescue bronchodilator) FEV_1_ (Figure 2). Similar results were obtained for montelukast 10 mg QD compared with placebo. However, mean difference in change from baseline between FP 100 μg BID and placebo achieved nominal statistical significance (Figure 2). The repeated measures sensitivity analysis of change from baseline in trough FEV_1_ indicated an overall increase for all doses of GSK2190915 (Figure 3) with the greatest increase observed for FP 100 μg BID. With the repeated measures analysis, treatment differences (active minus placebo) achieved nominal statistical significance for GSK2190915 30 mg QD at Week 8 (least squares [LS] mean difference: 0.115 L [95% confidence interval, CI: 0.00, 0.23], p = 0.044). For FP 100 μg BID, nominal statistical significance (active minus placebo) was achieved at all time-points (Weeks 1 to 8).

There were no statistically significant differences for any dose of GSK2190915 versus placebo in the secondary endpoints of morning and evening PEF, symptom-free days and nights, rescue-free days and nights, night-time symptom scores and night-time SABA use (Additional file 2); similar results were observed for montelukast 10 mg QD versus placebo. However, there were nominally statistically significant differences for FP 100 μg versus placebo for symptom-free days and rescue-free days. Nominally statistically significant reductions were observed for comparisons between GSK2190915 30 mg QD and placebo for the secondary endpoints of day-time symptom scores and day-time SABA use (Additional file 2). Comparisons between montelukast 10 mg QD or FP 100 μg and placebo showed a nominally statistically significant decrease for day-time SABA use (Additional file 2). Total scores for ACQ and total and domain scores for AQLQ were similar for placebo, GSK2190915 (any dose) and montelukast 10 mg QD whilst a nominally statistically significant decrease was observed for FP 100 μg versus placebo (Additional file 2). The percentage of symptom-free 24-hour periods, rescue-free 24-hour periods and 24-hour period symptom scores was similar for placebo, GSK2190915 (any dose) and montelukast 10 QD. However, the percentage of symptom-free 24-hour periods and rescue-free 24-hour periods was nominally statistically significantly reduced for FP 100 μg compared with placebo. There were nominally statistically significant decreases for GSK2190915 30 mg QD, montelukast 10 mg QD and FP 100 μg versus placebo for 24-hour SABA use. Notable improvements were observed in the placebo group for all efficacy endpoints.

### Pharmacokinetics/pharmacodynamics results

Plasma GSK2190915 levels increased in an approximately dose proportional manner. Geometric mean (inter-subject variability [%CV]) trough plasma GSK2190915 concentrations were 29 (73) ng/mL following GSK2190915 10 mg QD, 66 (84) ng/mL following GSK2190915 30 mg QD, 157 (110) ng/mL following GSK2190915 100 mg QD, and 370 (111) ng/mL following GSK2190915 300 mg QD. The sparse plasma GSK2190915 concentration-time data were described by a one-compartment population pharmacokinetic model. Population mean (%CV) oral clearance was 16 (76) L/hour and apparent volume of distribution was 162 (99) L. Model derived geometric mean (%CV) area under the plasma GSK2190915 concentration curve at steady-state was 761 (72) ng.hour/mL for GSK2190915 10 mg QD, 2197 (75) ng.hour/mL for GSK2190915 30 mg QD, 6390 (64) ng.hour/mL for GSK2190915 100 mg QD and 19315 (88) ng.hour/mL for GSK2190915 300 mg QD.

**Figure 4:**
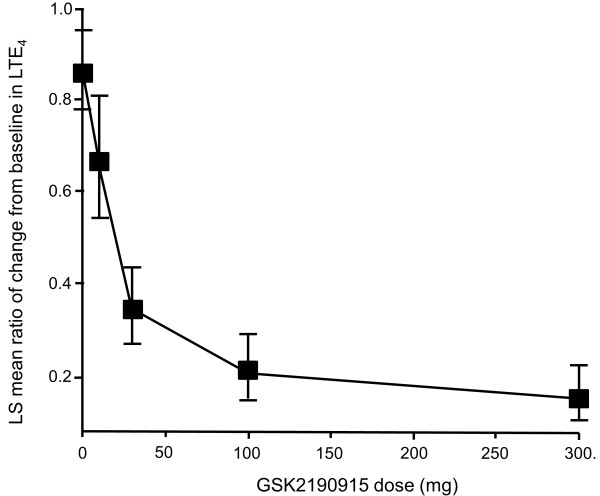
**Dose–response plot of urinary LTE**_**4**_**.** Ratio of mean change from baseline in urinary LTE_4_. Error bars represent 95% confidence interval. LS = least squares.

Exposure to GSK2190915 was associated with a decrease in urinary LTE_4_ levels (Figure 4). The plasma GSK2190915 concentration versus urinary LTE_4_ relationship was described by an inhibitory E_max_ model. The GSK2190915 plasma concentration associated with 50% of maximum (100%) urinary LTE_4_ inhibition was 33 (113) ng/mL, which equates to a GSK2190915 trough plasma concentration achieved with a dose of 10 mg.

### Safety and tolerability

The incidence of adverse events was generally similar between treatment groups (Table 2). The most frequently reported adverse events were headache and nasopharyngitis. Four subjects experienced serious adverse events: dislocated joint (n = 1, GSK2190915 10 mg QD), cranial neuritis (n = 1, GSK2190915 30 mg QD), small intestine obstruction (n = 1, GSK2190915 100 mg QD) and injured cartilage (n = 1, FP 100 μg BID); none of the events was considered related to study drug by the investigators and all resolved except the cranial neuritis, which led to subject withdrawal from the study. Eleven subjects were discontinued from the study as a consequence of an adverse event. Five of these were considered drug-related: decreased appetite and nausea (n = 1, placebo), dyspnoea (n = 1, GSK2190915 10 mg QD), extrasystoles and diarrhoea (n = 2, GSK2190915 300 mg QD) and drug eruption (n = 1, montelukast 10 mg QD). No drug-related adverse events were reported by more than one subject in any treatment group. Fifteen subjects experienced asthma exacerbations across all treatment groups except FP 100 μg BID; all episodes were treated with corticosteroids and all exacerbations resolved. One subject became pregnant during the run-in period and was withdrawn before randomisation. No abnormalities in male reproductive health were detected in the follow-up data from the male subjects who participated in the study and underwent follow-up testing. There were no significant or clinically meaningful differences between active treatments and placebo in blood chemistry (including liver function), haematology, urinalysis, vital signs and ECG parameters.

**Table 2:** Summary of most frequently reported adverse events (>1% of all subjects who received GSK2190915)

	**Placebo**	**GSK2190915 10 mg QD**	**GSK2190915 30 mg QD**	**GSK2190915 100 mg QD**	**GSK2190915 300 mg QD**	**Total GSK2190915**	**FP 100 μg BID**	**Montelukast 10 mg QD**
	**N = 100**	**N = 99**	**N = 100**	**N = 100**	**N = 101**	**N = 400**	**N = 103**	**N = 97**
	**n (%)**	**n (%)**	**n (%)**	**n (%)**	**n (%)**	**n (%)**	**n (%)**	**n (%)**
Any event	20 (20)	25 (25)	20 (20)	20 (20)	24 (24)	89 (22)	25 (24)	24 (25)
Headache	3 (3)	7 (7)	2 (2)	4 (4)	4 (4)	17 (4)	9 (9)	9 (9)
Nasopharyngitis	5 (5)	6 (6)	3 (3)	2 (2)	5 (5)	16 (4)	5 (5)	5 (5)
Cough	1 (1)	2 (2)	2 (2)	2 (2)	2 (2)	8 (2)	3 (3)	1 (1)
Nausea	1 (1)	2 (2)	1 (1)	1 (1)	3 (3)	7 (2)	1 (<1)	0
Oropharyngeal pain	1 (1)	1 (1)	0	2 (2)	3 (3)	6 (2)	1 (<1)	1 (1)
Back pain	0	1 (1)	2 (2)	2 (2)	1 (<1)	6 (2)	0	2 (2)

*QD* Once-daily, *FP* Fluticasone propionate, *BID* Twice-daily.

## Discussion

Administration of GSK2190915 for 8 weeks led to numerical increases in mean change from baseline in trough FEV_1_ at all doses, although none of these achieved statistical significance compared with placebo. A repeated measures sensitivity analysis of the primary endpoint suggested nominal statistical significance for GSK2190915 30 mg compared with placebo. Improvements were observed in mean day-time symptom scores and mean day-time SABA use for GSK2190915 30 mg versus placebo. No additional benefit was gained by administration of GSK2190915 doses greater than 30 mg. The results obtained in this study suggest that a GSK2190915 dose of 10 mg QD may be sub-optimal and furthermore suggest that the FEV_1_ dose–response across the range investigated (30–300 mg) was markedly flat. This was in contrast to the plasma GSK2190915 concentrations that showed increases with dose, and the urinary LTE_4_ levels that decreased in a dose/GSK2190915 plasma concentration-dependent manner. These pharmacokinetic and pharmacodynamic observations were associated with high inter-subject variability but were comparable to previously reported clinical studies [18].

The results reported in this study population suggest that inhibition of FLAP may not provide further improvement on efficacy endpoints beyond those achieved with cysLT receptor antagonism. However, this requires further evaluation in specifically designed clinical trials. In patients with severe asthma, where neutrophil inflammation plays a significant role in disease pathology, zileuton has demonstrated improvements in lung function. Although zileuton is the only LT synthesis inhibitor currently available, its use has been limited by the need to monitor hepatic enzyme levels and the high frequency of dosing [16]. The findings from the current study indicate that GSK2190915 could play a similar role to zileuton in terms of its anti-LT production activity.

Urinary LTE_4_ levels decreased in a manner dependent on GSK2190915 dose/GSK2190915 plasma concentration, confirming that GSK2190915 is capable of inhibiting the 5-LO pathway in subjects with persistent asthma. GSK2190915 achieved 50% of maximum estimated inhibition of urinary LTE_4_ at a plasma concentration of 33 ng/mL, which is comparable to the value estimated from earlier studies in healthy subjects (unpublished observations). However, reduced LTE_4_ was only associated with small and non-statistically significant changes in FEV_1_ from baseline. Thus, the measures of efficacy of the present study failed to demonstrate a dose–response for GSK2190915 on FEV_1_ even though the pharmacokinetic data confirmed that an increase in drug exposure was associated with a dose-dependent decrease in the levels of urinary LTE_4_.

Previous dose-ranging studies with anti-LT agents have also shown poor relationships between urinary LTE_4_ concentrations and changes in FEV_1_[20-22]. A study of MK-0633, a 5-LO inhibitor, showed that inhibition of urinary LTE_4_ of approximately 90% was associated with a relatively modest change in FEV_1_ from baseline: 0.20 L compared with 0.13 L for placebo in change from baseline over the last 4 weeks of a 6-week treatment period [21]. Zileuton led to significant increases in FEV_1_ after treatment for 4 weeks (change from baseline: 0.32 L; 95% CI: 0.16, 0.48) but the levels of inhibition of urinary LTE_4_ achieved were only 39% [22]. Therefore, the results obtained in our study where inhibition of urinary LTE_4_ was associated with a small change from baseline in FEV_1_ (0.13 L for placebo and 0.18–0.23 L for GSK2190915) are consistent with previous studies and support the hypothesis that changes in LTE_4_ and FEV_1_ may not always be highly correlated.

GSK2190915 was well-tolerated and there were no differences between treatment groups in the frequency of adverse events. Previous studies of zileuton and MK-0633 suggest that these molecules may have less than optimal safety and tolerability profiles as liver abnormalities were identified [16,21]. In one study with MK-0633, a planned extension to the study was terminated prematurely and the patients discontinued because of apparent dose-related increases in alanine aminotransferase and aspartate aminotransferase levels [21]. No such abnormalities were observed in the current study.

As the original protocol allowed recruitment of both male and female subjects, 56 males received at least one dose of study medication. However, interim histopathological assessments of the male reproductive tract from toxicology studies in rats, which were reported during the conduct of the study, revealed testicular toxicity at high exposure levels of GSK2190915. In a proportion of the rats receiving the maximum feasible dose of GSK2190915 (1000 mg/kg/day), testicular atrophy and hypospermia in the epididymides were observed. The reasons behind this finding were unclear at the time and, therefore, it was decided to withdraw all males and only recruit female subjects for the remainder of the study. Although the study population was predominantly female (92%), the results obtained in the study appear applicable to both genders as, generally, no gender-specific results have been described in asthma treatment.

There is no clear reason that can explain why subjects in the placebo group demonstrated such a high response in the primary and secondary efficacy endpoints. Although there were improvements within active treatment groups, the placebo response may have limited the opportunity for demonstrating significant changes in the efficacy endpoints for active treatments relative to placebo. It is possible that, as the study population was comprised of subjects with relatively mild asthma controlled with a SABA, some subjects improved spontaneously and others are likely to have benefited from the frequent contact with clinical staff and the greater attention received, which may be particularly relevant in a mild asthmatic population.

It could be questioned why no statistically significant difference between GSK2190915 and placebo was achieved for the primary efficacy endpoint, particularly as the outcome measure defined was the most appropriate for a dose range study in asthma. The observed response to FP, the positive control, indicated that the enrolled population was capable of improvement in terms of their lung function response. Furthermore, the study was well designed, incorporated a relatively wide dose range and active comparator and placebo arms were included in the design. It seems to be the case that there are areas in which the design of a randomised trial may result in gaps in the evidence they collect [23]. In particular, randomised trials include tightly controlled and well-characterised populations, tend to be limited in the number of outcomes evaluated and are relatively short in duration [23]. For chronic conditions such as asthma, concerns exist regarding the external validity of the data gathered in randomised trials and the ability to extrapolate these data to the heterogeneous patient population treated in everyday clinical practice.

## Conclusions

In subjects with persistent asthma, efficacy was demonstrated for GSK2190915 30 mg compared with placebo in mean day-time symptom scores and mean day-time SABA use. No additional improvement on efficacy endpoints was gained by administration of GSK2190915 doses greater than 30 mg. GSK2190915 was associated with a clear and dose-dependent reduction in urinary LTE_4_. However, this did not translate into a statistically significant improvement in mean trough FEV_1_ at Week 8 compared with placebo, although the presence of a notable placebo response may have hindered the assessment of the response to active treatment. The effect of GSK2190915 for primary and secondary efficacy endpoints was comparable with montelukast but less than low dose FP. GSK2190915 was generally safe and well-tolerated. The GSK2190915 30 mg dose appears to be the most appropriate for study in future clinical trials.

## Competing interest

Richard MA Follows, Shu-Yen Ho, Claire L Ambery, and Barbara A McQuade are GlaxoSmithKline employees. Neil G Snowise and Kevin Smart were employed by GlaxoSmithKline at the time the study was conducted.

## Authors’ contribution

RMAF, SYH and CLA participated in the conception and design of the study, and in the analysis and interpretation of the data. NGS and BAM contributed to the conduct of the study, protocol amendment and the analysis and interpretation of the data. KS contributed to analysis and interpretation of the data. All authors have made critical revisions of draft versions of the manuscript and approved the final manuscript.

## Supplementary Material

Additional file 1 **Study treatments administered.** Table detailing the study treatments administered following the double-dummy design. Click here for file

Additional file 2 **Summary of secondary efficacy parameters.** Summary of the analysis of the secondary efficacy endpoints of the study. Click here for file
