# Supplementary material for: Efficacy, safety and tolerability of GSK2190915, a 5-lipoxygenase activating protein inhibitor, in adults and adolescents with persistent asthma: a randomised dose-ranging study
Source: Respir Res. 2013 May 17;14(1):54. doi: 10.1186/1465-9921-14-54 (PMC3732081; doi:10.1186/1465-9921-14-54)
Supplement: Additional file 1 — Study treatments administered. Table detailing the study treatments administered following the double-dummy design. [file 1465-9921-14-54-S1.docx]

Additional file 1. Study treatments administered

| Treatment Arm | Morning | Evening |
| --- | --- | --- |
| GSK2190915  10 mg | 1 x 10 mg GSK2190915 tablet  1 x placebo tablet  1 x placebo DISKUS/ACCUHALER inhalation | 1 x placebo DISKUS/ACCUHALER inhalation  1 x placebo capsule |
| GSK2190915  30 mg | 1 x 30 mg GSK2190915 tablet  1 x placebo tablet  1 x placebo DISKUS/ACCUHALER inhalation | 1 x placebo DISKUS/ACCUHALER inhalation  1 x placebo capsule |
| GSK2190915  100 mg | 1 x 100 mg GSK2190915 tablet  1 x placebo tablet  1 x placebo DISKUS/ACCUHALER inhalation | 1 x placebo DISKUS/ACCUHALER inhalation  1 x placebo capsule |
| GSK2190915  300 mg | 1 x 100 mg GSK2190915 tablet  1 x 200 mg GSK2190915 tablet  1 x placebo DISKUS/ACCUHALER inhalation | 1 x placebo DISKUS/ACCUHALER inhalation  1 x placebo capsule |
| FP  100 µg | 1 x 100 µg DISKUS/ACCUHALER inhalation  2 x placebo tablets | 1 x 100 µg DISKUS/ACCUHALER inhalation  1 x placebo capsule |
| Montelukast  10 mg | 2 x placebo tablets  1 x placebo DISKUS/ACCUHALER inhalation | 1 x 10 mg montelukast capsule  1 x placebo DISKUS/ACCUHALER inhalation |
| Placebo | 2 x placebo tablets  1 x placebo DISKUS/ACCUHALER inhalation | 1 x placebo DISKUS/ACCUHALER inhalation  1 x placebo capsule |
