# Supplementary material for: Efficacy, safety and tolerability of GSK2190915, a 5-lipoxygenase activating protein inhibitor, in adults and adolescents with persistent asthma: a randomised dose-ranging study
Source: Respir Res. 2013 May 17;14(1):54. doi: 10.1186/1465-9921-14-54 (PMC3732081; doi:10.1186/1465-9921-14-54)
Supplement: Additional file 2 — Summary of secondary efficacy parameters. Summary of the analysis of the secondary efficacy endpoints of the study. [file 1465-9921-14-54-S2.docx]

Additional file 2. Summary of secondary efficacy parameters

| Treatment | Baseline, mean (SD) | N | Change from baseline Weeks 1–8, mean (SD) | Change from baseline Weeks 1–8, LS mean difference (95% CI) | | N | p-value vs placebo |
| --- | --- | --- | --- | --- | --- | --- | --- |
| **Evening peak expiratory flow (L/min)** | | | | | | | |
| Placebo | 320.29 (99.84) | 100 | 8.05 (33.19) | – | | – | – |
| GSK2190915 10 mg QD | 313.03 (98.86) | 99 | 8.76 (33.71) | -0.39 (-9.25, 8.48) | | 99 | NS |
| GSK2190915 30 mg QD | 298.85 (94.05) | 100 | 11.05 (34.90) | 1.37 (-7.50, 10.23) | | 99 | NS |
| GSK2190915 100 mg QD | 316.38 (93.74) | 100 | 6.43 (28.02) | -1.80 (-10.62, 7.03) | | 100 | NS |
| GSK2190915 300 mg QD | 323.76 (98.30) | 101 | 9.65 (35.59) | 2.32 (-6.49, 11.13) | | 101 | NS |
| FP 100 µg BID | 320.81 (90.15) | 103 | 10.31 (34.84) | 2.46 (-6.36, 11.28) | | 101 | NS |
| Montelukast 10 mg QD | 335.88 (99.01) | 97 | 6.26 (29.45) | 0.53 (-8.40, 9.45) | | 97 | NS |
| **Morning peak expiratory flow (L/min)** | | | | | | | |
| Placebo | 305.86 (98.76) | 100 | 11.71 (31.87) | – | | – | – |
| GSK2190915 10 mg QD | 296.59 (97.81) | 99 | 14.90 (29.59) | 1.46 (-7.65, 10.58) | | 99 | NS |
| GSK2190915 30 mg QD | 284.44 (91.04) | 100 | 17.28 (36.03) | 3.75 (-5.36, 12.87) | | 99 | NS |
| GSK2190915 100 mg QD | 305.78 (93.44) | 100 | 8.32 (28.66) | -3.05 (-12.12, 6.03) | | 100 | NS |
| GSK2190915 300 mg QD | 305.20 (94.98) | 101 | 16.12 (34.14) | 4.59 (-4.47, 13.64) | | 101 | NS |
| FP 100 µg BID | 309.36 (88.64) | 103 | 14.60 (39.00) | 3.48 (-5.61, 12.58) | | 100 | NS |
| Montelukast 10 mg QD | 316.97 (90.52) | 97 | 15.26 (37.09) | 5.62 (-3.55, 14.78) | | 97 | NS |
| **Percentage symptom-free days** | | | | | | | |
| Placebo | 7.98 (16.02) | 100 | 14.35 (26.34) | – | | – | – |
| GSK2190915 10 mg QD | 10.52 (21.10) | 99 | 15.17 (31.21) | 1.17 (-6.73, 9.07) | | 99 | NS |
| GSK2190915 30 mg QD | 7.91 (17.63) | 100 | 18.57 (27.86) | 4.56 (-3.33, 12.45) | | 99 | NS |
| GSK2190915 100 mg QD | 7.14 (20.79) | 100 | 15.86 (30.83) | 1.33 (-6.54, 9.19) | | 100 | NS |
| GSK2190915 300 mg QD | 7.64 (19.29) | 101 | 14.37 (24.34) | 0.08 (-7.76, 7.93) | | 101 | NS |
| FP 100 µg BID | 11.56 (22.01) | 103 | 21.75 (31.85) | 8.20 (0.34, 16.07) | | 101 | ≤0.05 |
| Montelukast 10 mg QD | 11.18 (19.61) | 97 | 16.00 (29.12) | 2.89 (-5.05, 10.83) | | 97 | NS |
| **Percentage of symptom-free nights** | | | | | | | |
| Placebo | 18.72 (29.74) | 100 | 14.15 (31.08) | – | | – | – |
| GSK2190915 10 mg QD | 16.96 (26.75) | 99 | 15.86 (29.77) | 0.84 (-7.22, 8.89) | | 99 | NS |
| GSK2190915 30 mg QD | 19.67 (31.23) | 100 | 16.23 (26.49) | 2.72 (-5.33, 10.76) | | 99 | NS |
| GSK2190915 100 mg QD | 19.50 (32.94) | 100 | 16.07 (32.16) | 2.12 (-5.90, 10.14) | | 100 | NS |
| GSK2190915 300 mg QD | 19.28 (30.07) | 101 | 12.08 (29.00) | -1.78 (-9.78, 6.22) | | 101 | NS |
| FP 100 µg BID | 18.53 (28.57) | 103 | 20.18 (33.22) | 5.95 (-2.08, 13.98) | | 100 | NS |
| Montelukast 10 mg QD | 20.33 (28.68) | 97 | 18.61 (28.54) | 5.39 (-2.69, 13.48) | | 97 | NS |
| **Percentage of rescue-free days** | | | | | | | |
| Placebo | 18.15 (29.32) | 100 | 16.85 (29.84) | – | | – | – |
| GSK2190915 10 mg QD | 17.07 (27.63) | 99 | 23.95 (34.47) | 6.11 (-2.50, 14.71) | | 99 | NS |
| GSK2190915 30 mg QD | 17.38 (28.72) | 100 | 21.45 (28.65) | 4.11 (-4.49, 12.70) | | 99 | NS |
| GSK2190915 100 mg QD | 20.48 (31.55) | 100 | 18.59 (33.62) | 2.15 (-6.42, 10.72) | | 100 | NS |
| GSK2190915 300 mg QD | 20.99 (34.16) | 101 | 17.95 (31.49) | 1.71 (-6.84, 10.26) | | 101 | NS |
| FP 100 µg BID | 21.08 (30.61) | 103 | 26.26 (35.13) | 9.59 (1.03, 18.15) | | 101 | ≤0.05 |
| Montelukast 10 mg QD | 19.33 (29.29) | 97 | 22.99 (32.20) | 6.75 (-1.89, 15.38) | | 97 | NS |
| **Percentage of rescue-free nights** | | | | | | | |
| Placebo | 24.78 (33.95) | 100 | 17.71 (29.95) | – | | – | – |
| GSK2190915 10 mg QD | 27.32 (34.18) | 99 | 19.79 (34.77) | 2.35 (-6.09, 10.78) | | 99 | NS |
| GSK2190915 30 mg QD | 27.68 (35.26) | 100 | 17.43 (27.80) | 0.43 (-8.00, 8.86) | | 99 | NS |
| GSK2190915 100 mg QD | 28.96 (35.70) | 100 | 19.32 (34.08) | 2.70 (-5.70, 11.11) | | 100 | NS |
| GSK2190915 300 mg QD | 29.39 (35.96) | 101 | 15.23 (33.79) | -1.22 (-9.60, 7.17) | | 101 | NS |
| FP 100 µg BID | 27.20 (34.63) | 103 | 24.91 (34.39) | 7.49 (-0.92, 15.90) | | 100 | NS |
| Montelukast 10 mg QD | 30.80 (37.17) | 97 | 19.47 (31.28) | 3.61 (-4.87, 12.09) | | 97 | NS |
| **Day-time symptom scores** | | | | | | | |
| Placebo | 1.64 (0.71) | 100 | -0.37 (0.58) | – | | – | – |
| GSK2190915 10 mg QD | 1.44 (0.67) | 99 | -0.31 (0.64) | -0.01 (-0.17, 0.16) | | 99 | NS |
| GSK2190915 30 mg QD | 1.63 (0.72) | 100 | -0.52 (0.69) | -0.17 (-0.33, -0.01) | | 99 | ≤0.05 |
| GSK2190915 100 mg QD | 1.64 (0.75) | 100 | -0.40 (0.74) | -0.03 (-0.19, 0.13) | | 100 | NS |
| GSK2190915 300 mg QD | 1.50 (0.71) | 101 | -0.32 (0.55) | -0.00 (-0.16, 0.16) | | 101 | NS |
| FP 100 µg BID | 1.49 (0.78) | 103 | -0.41 (0.67) | -0.10 (-0.26, 0.06) | | 101 | NS |
| Montelukast 10 mg QD | 1.54 (0.68) | 97 | -0.40 (0.52) | -0.08 (-0.24, 0.09) | | 97 | NS |
| **Night-time symptom scores** | | | | | | | |
| Placebo | 1.17 (0.63) | 100 | -0.23 (0.49) | – | | – | – |
| GSK2190915 10 mg QD | 1.17 (0.61) | 99 | -0.21 (0.52) | 0.03 (-0.11, 0.17) | | 99 | NS |
| GSK2190915 30 mg QD | 1.22 (0.70) | 100 | -0.33 (0.59) | -0.10 (-0.24, 0.04) | | 99 | NS |
| GSK2190915 100 mg QD | 1.24 (0.74) | 100 | -0.27 (0.63) | -0.02 (-0.16, 0.12) | | 100 | NS |
| GSK2190915 300 mg QD | 1.17 (0.71) | 101 | -0.22 (0.56) | 0.01 (-0.13, 0.15) | | 101 | NS |
| FP 100 µg BID | 1.23 (0.75) | 103 | -0.30 (0.57) | -0.05 (-0.19, 0.09) | | 100 | NS |
| Montelukast 10 mg QD | 1.15 (0.66) | 97 | -0.30 (0.44) | -0.08 (-0.22, 0.06) | | 97 | NS |
| **Day-time SABA use (inhalations)** | | | | | | | |
| Placebo | 1.74 (1.10) | 100 | -0.43 (0.85) | – | | – | – |
| GSK2190915 10 mg QD | 1.83 (1.10) | 99 | -0.60 (0.88) | -0.13 (-0.33, 0.07) | | 99 | NS |
| GSK2190915 30 mg QD | 1.86 (1.33) | 100 | -0.74 (0.96) | -0.26 (-0.47, -0.06) | | 99 | ≤0.05 |
| GSK2190915 100 mg QD | 1.65 (1.28) | 100 | -0.48 (0.87) | -0.08 (-0.28, 0.13) | | 100 | NS |
| GSK2190915 300 mg QD | 1.52 (0.97) | 101 | -0.39 (0.69) | -0.05 (-0.25, 0.16) | | 101 | NS |
| FP 100 µg BID | 1.60 (1.00) | 103 | -0.63 (0.91) | -0.25 (-0.45, -0.04) | | 101 | ≤0.05 |
| Montelukast 10 mg QD | 1.75 (1.21) | 97 | -0.63 (0.76) | -0.21 (-0.41, -0.00) | | 97 | 0.05 |
| **Night-time SABA use (inhalations)** | | | | | | | |
| Placebo | 1.35 (0.85) | 100 | -0.29 (0.66) | – | | – | – |
| GSK2190915 10 mg QD | 1.45 (0.98) | 99 | -0.43 (0.71) | -0.10 (-0.28, 0.09) | | 99 | NS |
| GSK2190915 30 mg QD | 1.43 (1.21) | 100 | -0.46 (0.81) | -0.14 (-0.32, 0.05) | | 99 | NS |
| GSK2190915 100 mg QD | 1.35 (1.15) | 100 | -0.42 (0.81) | -0.12 (-0.31, 0.06) | | 100 | NS |
| GSK2190915 300 mg QD | 1.29 (1.04) | 101 | -0.28 (0.78) | -0.00 (-0.19, 0.18) | | 101 | NS |
| FP 100 µg BID | 1.34 (0.91) | 103 | -0.46 (0.81) | -0.17 (-0.36, 0.01) | | 100 | NS |
| Montelukast 10 mg QD | 1.36 (1.16) | 97 | -0.46 (0.71) | -0.16 (-0.34, 0.03) | | 97 | NS |
| **Asthma quality of life questionnaire, total score** | | | | | | | |
| Placebo | 4.54 (0.89) | 100 | 0.51 (0.98) | – | – | | – |
| GSK2190915 10 mg QD | 4.53 (1.03) | 99 | 0.63 (1.07) | 0.13 (-0.12, 0.38) | 99 | | NS |
| GSK2190915 30 mg QD | 4.49 (0.94) | 100 | 0.63 (1.04) | 0.11 (-0.14, 0.36) | 100 | | NS |
| GSK2190915 100 mg QD | 4.57 (0.94) | 100 | 0.51 (1.03) | 0.02 (-0.23, 0.27) | 100 | | NS |
| GSK2190915 300 mg QD | 4.52 (1.04) | 101 | 0.65 (1.01) | 0.13 (-0.12, 0.38) | 101 | | NS |
| FP 100 µg BID | 4.58 (1.00) | 103 | 0.86 (1.03) | 0.34 (0.10, 0.59) | 103 | | ≤0.05 |
| Montelukast 10 mg QD | 4.63 (0.87) | 97 | 0.54 (0.90) | 0.11 (-0.14, 0.36) | 97 | | NS |
| **Asthma control questionnaire, total score** | | | | | | | |
| Placebo | 2.39 (0.58) | 100 | -0.43 (0.89) | – | – | | – |
| GSK2190915 10 mg QD | 2.42 (0.64) | 99 | -0.61 (0.86) | -0.17 (-0.42, 0.07) | 99 | | NS |
| GSK2190915 30 mg QD | 2.42 (0.64) | 100 | -0.57 (0.93) | -0.15 (-0.39, 0.09) | 100 | | NS |
| GSK2190915 100 mg QD | 2.35 (0.65) | 100 | -0.55 (0.89) | -0.18 (-0.42, 0.05) | 100 | | NS |
| GSK2190915 300 mg QD | 2.34 (0.70) | 101 | -0.48 (0.94) | -0.09 (-0.33, 0.15) | 101 | | NS |
| FP 100 µg BID | 2.39 (0.71) | 103 | -0.96 (0.93) | -0.53 (-0.77, -0.29) | 103 | | <0.001 |
| Montelukast 10 mg QD | 2.25 (0.67) | 97 | -0.54 (0.78) | -0.22 (-0.46, 0.02) | 97 | | NS |
| SD = standard deviation; CI = confidence interval; FEV_1_ = forced expiratory volume in 1 second; QD = once-daily; NS = not significant; FP = fluticasone propionate; BID = twice-daily; SABA = short-acting beta2-agonist | | | | | | | |
